# Pediatric Central Retinal Artery Occlusion in a Patient With Elevated Lipoprotein (A) and Factor VIII Levels

**DOI:** 10.7759/cureus.76258

**Published:** 2024-12-23

**Authors:** Richard Yi, Ryan D Gabbard, Tate Saurey, Saad Al-Kadhi, Stephen Hypes, Barron Fishburne

**Affiliations:** 1 Ophthalmology, University of South Carolina/Prisma Health, Columbia, USA; 2 Ophthalmology, Palmetto Retina Center, Columbia, USA

**Keywords:** central retinal artery occlusion, factor viii, lipoprotein (a), pediatric, retina artery occlusion

## Abstract

The purpose of this manuscript is to report a rare case of pediatric central retinal artery occlusion (CRAO) in the setting of atypical hypercoagulable tests. An 11-year-old female presented to the emergency department with painless, visual changes in the left eye. Ophthalmological examination was remarkable for a central area of retinal ischemia and edema with sparing along the distribution of the cilioretinal artery along with a cherry red spot, all of which were consistent with a CRAO. Neuroimaging and infectious disease work-up were unremarkable. Hematological labs demonstrated elevated lipoprotein (a) and factor VIII activity levels. CRAOs generally occur in the adult population in the setting of cardiovascular risk factors and are uncommon in the pediatric age group. In such a demographic, additional investigation including hypercoagulable work-up is warranted. Elevated lipoprotein (a) is thought to be an independent risk factor for atherosclerosis while factor VIII levels can correlate with thromboemboli in the venous vasculature. Becoming aware of such risk factors can help guide the clinician with the diagnostic approach and management when presented with a similar pediatric case.

## Introduction

Central retinal artery occlusions (CRAOs) rarely occur in the pediatric population. Of these cases, hypercoagulable states and emboli are the most frequent etiologies. We present an 11-year-old healthy female with sudden, painless vision loss in the setting of elevated factor VIII and lipoprotein (a) levels. Elevated levels of factor VIII are an independent risk factor in the development of venous thromboemboli; however, they are not typically associated with emboli involving the arterial vasculature [1]. Elevated lipoprotein (a) levels have been demonstrated to enhance the risk of cardiovascular events; however, they are uncommon in the pediatric age group [2]. The collection and evaluation of protected patient health information of this patient were Health Insurance Portability and Accountability Act (HIPAA) compliant. This case report also adhered to the ethical principles outlined in the Declaration of Helsinki as amended in 2013.

## Case presentation

An 11-year-old female presented to the emergency department for left eye visual disturbances consisting of decreased vision and floaters. As per the family, the patient did not have any previous eye issues. She reported that her visual symptoms have been ongoing for roughly 24 hours and described it as tunnel-like vision seeing through “a box” in the center. She denied any photopsias or eye pain. Ophthalmic history was significant for wearing glasses but otherwise was unremarkable. She denied any history of eye trauma, amblyopia, strabismus, or procedures in the past. In addition, she denied any flu-like illnesses, recent travel, or recent contact with any pets such as cats. Of note, the patient was at a water park prior to the visual changes earlier in the week and reported riding on multiple high-intensity rides. 

**Figure 1:**
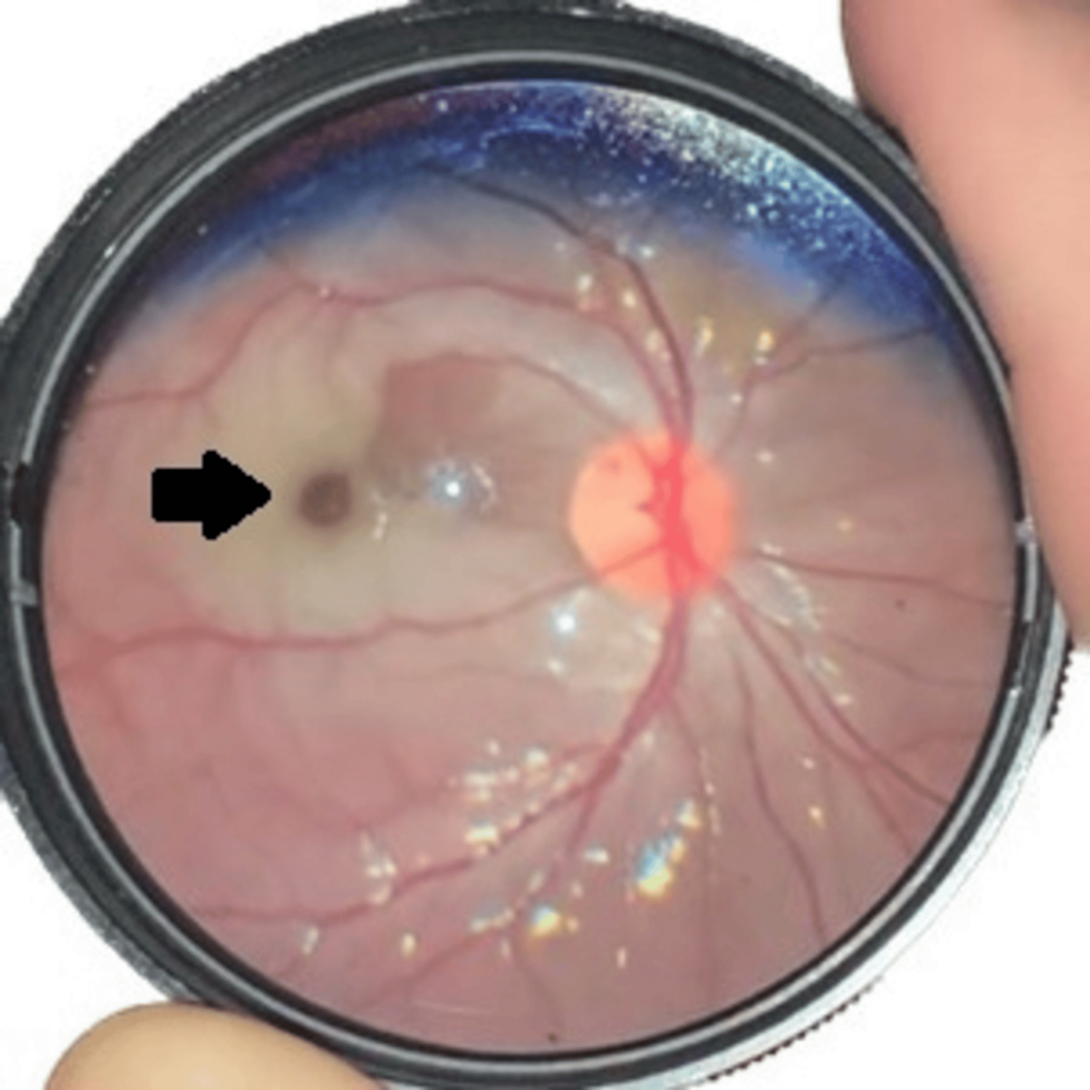
Photo of the patient’s left eye under a 20-diopter lens obtained with a handheld camera within the emergency department setting. As noted, the cherry-red spot (black arrow) can be visualized surrounded by retinal ischemia with sparing along the cilioretinal pathway.

On the exam, near visual acuity (NVA) was 20/20 in the right eye (OD) and 20/200 in the left eye (OS). Intraocular pressure was 17 in both eyes (OU). Color vision was 11/11 OD and 8/11 OS via Ishihara color plates; therefore, mildly reduced OS. Extraocular movements were normal in all directions. The slit lamp exam was normal. A dilated fundus exam (DFE) demonstrated normal-appearing optic nerves with a 0.2 c/d ratio and no evidence of nerve head edema. DFE OS demonstrated a central, small hyperpigmented retina surrounded by hypopigmented, edematous retina within the macula along with a small region of sparing along the cilioretinal vessel path (Figure 1). No embolus or thrombus was visualized in the retinal vasculature. 

Given the patient’s acute, unilateral painless vision loss and DFE findings consistent with a CRAO, she was admitted for thromboembolic work-up. Neuroimaging included MRI brain/orbits, MRA head/neck, pediatric echocardiogram, and vascular ultrasound, which were all unremarkable. Infectious labs such as syphilis, toxoplasmosis, and tuberculosis were unremarkable. Cholesterol level was elevated at 184 mg/dL (reference normal <170 mg/dL) along with high lipoprotein (a) at 236.5 nmol/L (reference normal <75 nmol/L). Hematology was consulted and recommended a hypercoagulable work-up that included protein C, protein S, antithrombin III, prothrombin gene mutation, homocysteine, anticardiolipin, beta-2 glycoprotein, beta-2 glycoprotein, lipoprotein (a), and factor V Leiden. Factor VIII was elevated at 186% and antithrombin III to greater than 140%, otherwise unremarkable.

**Figure 2:**
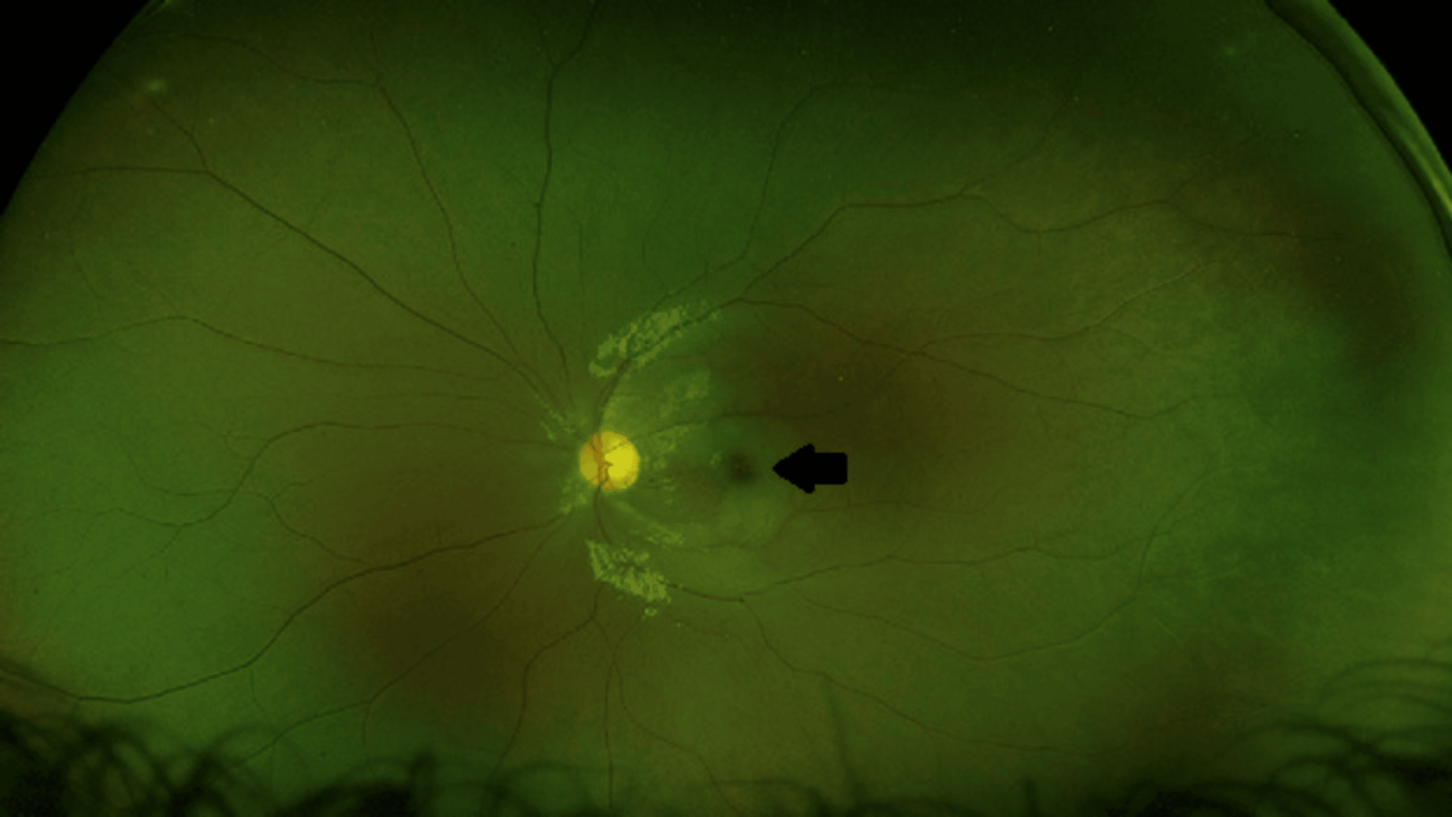
Fundus photo of the patient’s left eye, which demonstrate hypopigmented retinal ischemia within the macula and a hyperpigmented central foci (black arrow). Interestingly, the retina appears to be spared by such changes along the cilioretinal artery pathway. No evidence of emboli was noted on examination and images.

**Figure 3:**
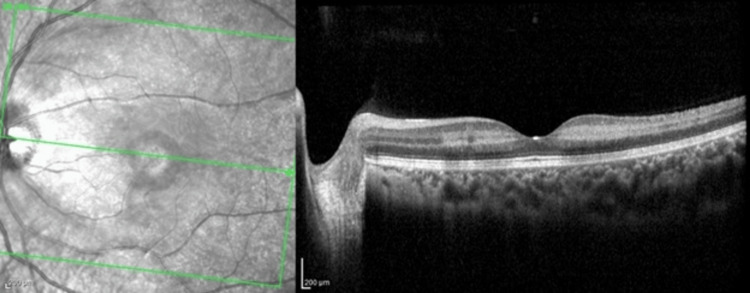
Macular optical coherence tomography (mOCT) of the patient’s affected left eye. The image reveals hyper-reflectivity of the inner retinal layers with edematous changes consistent with acute ischemia.

At the one-week follow-up visit, VA was stable and IOP remained within normal limits. SLE was negative for neovascularization of the iris. DFE OS demonstrated equivocal disc pallor with mild generalized arteriolar narrowing and cherry red spot sparing the inferonasal region (Figure 2). Macular optical coherence tomography (mOCT) was obtained that demonstrated hyperreflectivity of the inner retinal layers (Figure 3).

At the four-month follow-up visit, VA improved to 20/80 OS. DFE OS demonstrated 1+ optic disc pallor with regressed CRAO. The patient remained on aspirin 81 mg once daily as directed by her hematologist.

## Discussion

CRAO is a form of acute ischemic stroke that results in severe vision loss [3]. The incidence of CRAO in people under the age of 30 has been estimated at less than one in 50,000 [4]. Retinal artery occlusions are exceedingly rare in pediatric populations with most cases having a detectable etiology such as hypercoagulable states, emboli, migraine, infection, or trauma; however, some cases have been reported as idiopathic [5,6]. Of these causes, emboli have been found to be the most common, typically arising from the heart or carotid artery [7].

Hyperlipidemia is an atherosclerotic risk factor that has also been linked to CRAO, although more common in the adult population. In our case, lipoprotein (a) was significantly elevated with an otherwise unremarkable hypercoagulable work-up with the exception of factor VIII. Lipoprotein (a) has been demonstrated to have a high affinity for glycosaminoglycans of the human arterial wall to a greater extent than even low-density lipoproteins (LDLs) where it promotes inflammatory events that can propagate atheroma involvement and eventually arterial thrombosis [2]. There are limited case reports in the literature in regard to elevated lipoprotein levels and retinal artery occlusions. Coban-Karatas et al. reported a 13-year-old who developed a CRAO in the setting of hyperhomocysteinemia and elevated lipoprotein (a) [8]. In addition, Murata et al. demonstrated that patients with CRAO had significantly higher (p-value = 0.022) concentrations of lipoprotein (a) in comparison to the controls [9].

Elevated factor VIII levels are typically associated with occlusions of the venous vasculature that can lead to central retinal vein occlusions (CRVOs) or branch retinal vein occlusions (BRVOs); however, retinal artery occlusions are extremely rare. Chang et al. reported a 48-year-old female with elevated factor VIII levels found to have combined CRVO and CRAO [10]. One explanation of this phenomenon is Hayreh’s theory, which states that the lack of central retinal vein tributaries anterior to the occlusion after the occurrence of CRVO leads to a complete hemodynamic block of the retinal circulation causing a secondary CRAO [11]. In our case, there was no evidence of retinal vein occlusions, suggesting that the elevated factor VIII level may have been an acute phase reactant. 

Another underlying mechanism of action that we considered was trauma due to the patient being at a waterpark a few days prior to presentation to the ED. The logic is that traumatic injury to the region of interest results in a dissection or clot, which could be the source of the condition. With that said, however, magnetic resonance angiography (MRA) head and neck was unremarkable with no evidence of carotid abnormality. 

Management of CRAO is controversial and includes ocular massage, thrombolytic therapy, reduction of intraocular pressure via paracentesis or pharmacological measures, vasodilation of the central retinal artery, and hyperbaric oxygen. Except for ocular massage that can occasionally dislodge the embolus, there is no evidence that any of the rest have shown any benefit [11]. The purpose of this case report is to highlight a rare case of CRAO in the pediatric population in an otherwise healthy female with no known hypercoagulable conditions with the exception of hyperlipidemia. 

## Conclusions

In summary, we described a rare case of CRAO in a pediatric patient with elevated factor VIII and lipoprotein (a) levels. Previous reports in the literature have demonstrated the link between elevated lipoprotein (a) levels and increased cardiovascular risk. However, this is more common in adults with only a handful of pediatric cases reported. Despite their rare occurrence, pediatric CRAO should remain on a clinician's differential for painless, sudden vision loss, and a systemic evaluation should immediately be performed. 
